# Supplementary material for: Inflammation leads through PGE/EP 3 signaling to HDAC5/MEF2‐dependent transcription in cardiac myocytes
Source: EMBO Mol Med. 2018 Jun 15;10(7):e8536. doi: 10.15252/emmm.201708536 (PMC6034133; doi:10.15252/emmm.201708536)
Supplement: Supplementary file 1 — Appendix [file EMMM-10-e8536-s001.pdf]

Inflammation leads through PGE/EP$_3$ signaling to HDAC5/MEF2-dependent transcription in cardiac myocytes

András D. Tóth[1,2,*], Richard Schell[1,3,*], Magdolna Lévay[4], Christiane Vettel[4], Philipp Theis[1], Clemens Haslinger[1], Felix Alban[1], Stefanie Werhahn[1], Lina Frischbier[1], Jutta Krebs-Haupenthal[1], Dominique Thomas[5], Hermann-Josef Gröne[6], Metin Avkiran[7], Hugo A. Katus[3], Thomas Wieland[4,*] & Johannes Backs[1,*,#]

[1] Department of Molecular Cardiology and Epigenetics, Heidelberg University, Heidelberg, Germany; and DZHK (German Centre for Cardiovascular Research), partner site Heidelberg/Mannheim, Germany

[2] Department of Physiology, Faculty of Medicine, Semmelweis University, Budapest, Hungary

[3] Department of Cardiology, Heidelberg University, Germany; and DZHK (German Centre for Cardiovascular Research), partner site Heidelberg/Mannheim, Germany

[4] Experimental Pharmacology, European Center of Angioscience, Medical Faculty Mannheim, Heidelberg University, Mannheim, Germany; and DZHK (German Centre for Cardiovascular Research), partner site Heidelberg/Mannheim, Germany

[5] Institute of Clinical Pharmacology, Goethe-University Frankfurt, Frankfurt, Germany

[6] Department of Cellular and Molecular Pathology, German Cancer Research Center, Im Neuenheimer Feld 280, 69120 Heidelberg, Germany

[7] Cardiovascular Division, King's College London British Heart Foundation Centre of Research Excellence, The Rayne Institute, St Thomas' Hospital, London SE1 7EH, United Kingdom

* These authors contributed equally.

[#] Corresponding Author
Prof. Dr. med. Johannes Backs
Department Molecular Cardiology and Epigenetics
University Hospital Heidelberg
Im Neuenheimer Feld 669
69120 Heidelberg
Tel.: +49 6221 56-35271
Email: Johannes.backs@med.uni-heidelberg.de

Table of Content

Appendix Table S1 – Concentrations, descriptions and supporting references of the used compounds... 2

Appendix Table S2 – The used primer sequences for quantitative real-time PCR 3

References... 3

Appendix Table S1 – Concentrations, descriptions and supporting references of the used compounds.

Compound (used concentration)	Description with reference
**L798106 (200 nM)**	Potent and highly selective EP_3 receptor antagonist (Ki values are 0.3, 916, > 5000 and > 5000 nM at EP_3, EP_4, EP_1 and EP_2 receptors, respectively) (Clarke et al, 2004; Juteau et al, 2001).
**L161,982 (2 µM)**	Potent and selective EP_4 receptor antagonist (Ki values are 32, 7100, 5800, 6100 nM for rat EP_4, EP_3, EP_2 and EP_1 receptors, respectively) (Balzary et al, 2006; Machwate et al, 2001).
**AH6809 (10 µM)**	EP_1 (pA2 = 6.8) and EP_2 (Ki = 350 nM) receptor antagonist (Kiriyama et al, 1997).
**Bisindolylmaleimide I (3 µM)**	Selective inhibitor of protein kinase C. IC_{50} values are 0.0084, 0.0180, 0.210, 0.132, and 5.8 µM for α, β1, δ, ε and ζ PKC isoforms, respectively (Jacobson et al, 1995; Martiny-Baron et al, 1993).
**PTX (100 ng/ml)**	Bacterial toxin that inhibits $G_{i/o}$ and G_t via catalyzation of ADP-ribosylation (Vettel et al, 2012).
**NSC 23766 (100 µM)**	Selective inhibitor of Rac1 activation by preventing Rac1-GEF (TrioN and Tiam1, IC_{50} ~ 50 µM) interaction (Gao et al, 2004; Levay et al, 2013; Vettel et al, 2012).
**SQ 22536 (100 µM)**	Cell-permeable adenylate cyclase inhibitor (Harris et al, 1979; Mittra et al, 2006).
**AIP (1 µM)**	Selective and potent CaMKII inhibitor (IC_{50}: 40 nM) (Ishida et al, 1995).
**BPKDi (3 µM)**	Selective inhibitor of the three PKD isoforms with low nanomolar potency but it failed to inhibit members of the CaMKII and MARK families or SIK1 and GRK5 (Haworth et al, 2012; Meredith et al, 2010; Monovich et al, 2010).
**IPA-3 (30 µM)**	A selective small-molecule inhibitor that targets the autoregulatory mechanism of group 1 PAKs (PAK1,2,3) and promotes their inactive conformation. Group 1 PAKs were shown to be inhibited at 10 µM (Deacon et al, 2008; Vettel et al, 2012).

Appendix Table S2 – The used primer sequences for quantitative real-time PCR

Gene		Primer sequence
IL6	sense	5´-gatggatgctaccaaactggat-3´
	antisense	5´-ccaggtagctatggtactccaga-3´
TNFα	sense	5´-ctgtagcccacgtcgtagc-3´
	antisense	5´-ttgagatccatgccgttg-3´
Nur77	sense	5´-tgttgatgttcctgcctttg-3´
	antisense	5´-ggaggccatgtcgatcag-3´
Myomaxin	sense	5´-tctcctagaggcgctgtcc-3´
	antisense	5´-tgcacaagtctgattcttccat-3
AdamTS1	sense	5´-aaaggcattggctacttctttg-3´
	antisense	5´-ggactacagggagtgccatc-3´
ANP	sense	5´-cacagatctgatggatttcaaga-3´
	antisense	5´-cctcatcttctaccggcatc-3´
BNP	sense	5´-gtcagtcgcttgggctft-3´
	antisense	5´-ccagagctggggaaagaag-3´
18S	sense	5´-gcaattattccccatgaacg-3´
	antisense	5´-gggacttaatcaacgcaagc-3´

References

Balzary RW, Cocks TM (2006) Lipopolysaccharide induces epithelium- and prostaglandin E(2)-dependent relaxation of mouse isolated trachea through activation of cyclooxygenase (COX)-1 and COX-2. The Journal of pharmacology and experimental therapeutics 317**:** 806-812

Clarke DL, Giembycz MA, Patel HJ, Belvisi MG (2004) E-ring 8-isoprostanes inhibit ACh release from parasympathetic nerves innervating guinea-pig trachea through agonism of prostanoid receptors of the EP3-subtype. British journal of pharmacology 141**:** 600-609

Deacon SW, Beeser A, Fukui JA, Rennefahrt UE, Myers C, Chernoff J, Peterson JR (2008) An isoform-selective, small-molecule inhibitor targets the autoregulatory mechanism of p21-activated kinase. Chemistry & biology 15**:** 322-331

Gao Y, Dickerson JB, Guo F, Zheng J, Zheng Y (2004) Rational design and characterization of a Rac GTPase-specific small molecule inhibitor. Proceedings of the National Academy of Sciences of the United States of America 101**:** 7618-7623

Harris DN, Asaad MM, Phillips MB, Goldenberg HJ, Antonaccio MJ (1979) Inhibition of adenylate cyclase in human blood platelets by 9-substituted adenine derivatives. Journal of cyclic nucleotide research 5**:** 125-134

Haworth RS, Stathopoulou K, Candasamy AJ, Avkiran M (2012) Neurohormonal regulation of cardiac histone deacetylase 5 nuclear localization by phosphorylation-dependent and phosphorylation-independent mechanisms. Circulation research 110**:** 1585-1595

Ishida A, Kameshita I, Okuno S, Kitani T, Fujisawa H (1995) A novel highly specific and potent inhibitor of calmodulin-dependent protein kinase II. Biochemical and biophysical research communications 212**:** 806-812

Jacobson PB, Kuchera SL, Metz A, Schachtele C, Imre K, Schrier DJ (1995) Anti-inflammatory properties of Go 6850: a selective inhibitor of protein kinase C. The Journal of pharmacology and experimental therapeutics 275**:** 995-1002

Juteau H, Gareau Y, Labelle M, Sturino CF, Sawyer N, Tremblay N, Lamontagne S, Carriere MC, Denis D, Metters KM (2001) Structure-activity relationship of cinnamic acylsulfonamide analogues on the human EP3 prostanoid receptor. Bioorganic & medicinal chemistry 9**:** 1977-1984

Kiriyama M, Ushikubi F, Kobayashi T, Hirata M, Sugimoto Y, Narumiya S (1997) Ligand binding specificities of the eight types and subtypes of the mouse prostanoid receptors expressed in Chinese hamster ovary cells. British journal of pharmacology 122**:** 217-224

Levay M, Krobert KA, Wittig K, Voigt N, Bermudez M, Wolber G, Dobrev D, Levy FO, Wieland T (2013) NSC23766, a widely used inhibitor of Rac1 activation, additionally acts as a competitive antagonist at muscarinic acetylcholine receptors. The Journal of pharmacology and experimental therapeutics 347**:** 69-79

Machwate M, Harada S, Leu CT, Seedor G, Labelle M, Gallant M, Hutchins S, Lachance N, Sawyer N, Slipetz D et al (2001) Prostaglandin receptor EP(4) mediates the bone anabolic effects of PGE(2). Molecular pharmacology 60**:** 36-41

Martiny-Baron G, Kazanietz MG, Mischak H, Blumberg PM, Kochs G, Hug H, Marme D, Schachtele C (1993) Selective inhibition of protein kinase C isozymes by the indolocarbazole Go 6976. The Journal of biological chemistry 268**:** 9194-9197

Meredith EL, Beattie K, Burgis R, Capparelli M, Chapo J, Dipietro L, Gamber G, Enyedy I, Hood DB, Hosagrahara V et al (2010) Identification of potent and selective amidobipyridyl inhibitors of protein kinase D. J Med Chem 53**:** 5422-5438

Mittra S, Bourreau JP (2006) Gs and Gi coupling of adrenomedullin in adult rat ventricular myocytes. American journal of physiology Heart and circulatory physiology 290**:** H1842-1847

Monovich L, Vega RB, Meredith E, Miranda K, Rao C, Capparelli M, Lemon DD, Phan D, Koch KA, Chapo JA et al (2010) A novel kinase inhibitor establishes a predominant role for protein kinase D as a cardiac class IIa histone deacetylase kinase. FEBS Lett 584**:** 631-637

Vettel C, Wittig K, Vogt A, Wuertz CM, El-Armouche A, Lutz S, Wieland T (2012) A novel player in cellular hypertrophy: Gibetagamma/PI3K-dependent activation of the RacGEF TIAM-1 is required for alpha(1)-adrenoceptor induced hypertrophy in neonatal rat cardiomyocytes. Journal of molecular and cellular cardiology 53**:** 165-175
