# Supplementary material for: Inflammation leads through PGE/EP 3 signaling to HDAC5/MEF2‐dependent transcription in cardiac myocytes
Source: EMBO Mol Med. 2018 Jun 15;10(7):e8536. doi: 10.15252/emmm.201708536 (PMC6034133; doi:10.15252/emmm.201708536)
Supplement: Supplementary file 2 — Expanded View Figures PDF [file EMMM-10-e8536-s002.pdf]

Expanded View Figures

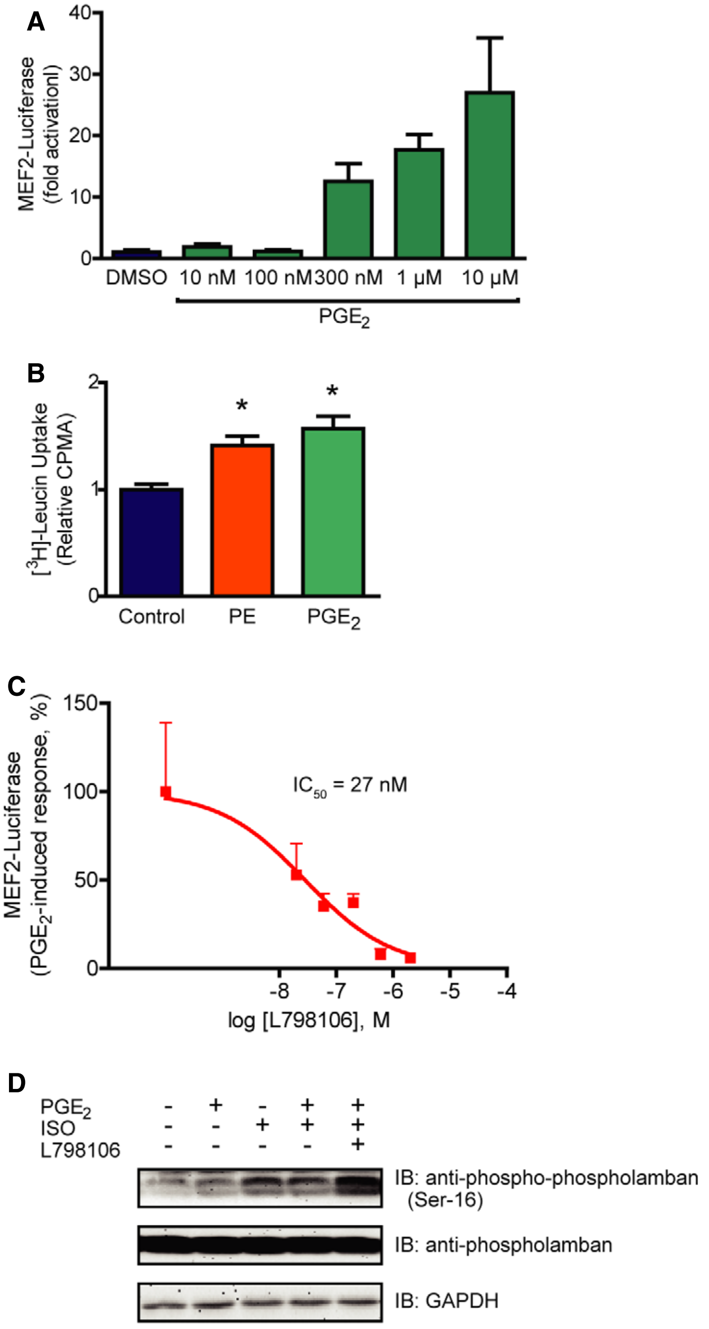

Figure EV1. PGE_2 activates MEF2 via the $G_{i/o}$ protein-coupled EP_3 receptor.

A Concentration dependency of the PGE_2-triggered MEF2 activity. MEF2 activity was assessed in 3xMEF2-Luc-infected NRVMs upon 24-h stimulation with increasing concentration of PGE_2.

B PGE_2 induces protein synthesis in NRVMs. After serum starvation, NRVMs were treated with media containing 1 μCi/ml [^{3}H]-leucine and 100 μM PE or 10 μM PGE_2 for 24 h. Thereafter, the cellular [^{3}H]-content was detected.

C Concentration-dependent effect of L798106 on the PGE_2-induced MEF2 activation. MEF2 activity was determined in 3xMEF2-Luc-expressing NRVMs pretreated with increasing concentration of L798106 for 20 min and treated with DMSO or 1 μM PGE2 for 24 h. The PGE_2-induced response was calculated by subtracting the MEF2-luciferase activity measured in vehicle-treated cells from the PGE_2-induced response in the case of all 798106 concentrations.

D L798106 inhibits the $G_{i/o}$ protein-coupled receptor of PGE_2. To prove that 798106 inhibits EP_3 receptor, a cAMP signal-decreasing receptor, we assessed the phosphorylation of phospholamban at a target site (Ser-16) of protein kinase A, a well-known cAMP-regulated kinase. NRVMs were pretreated with 200 nM L798106 for 20 min, then with 100 nM PGE_2 for 2 min. The β-adrenergic receptor agonist isoproterenol (ISO, 10 nM) was used to induce a cAMP signal. Phosphorylation of phospholamban was detected by immunoblot. In the presence of the EP3 receptor inhibitor, PGE_2 elevated the ISO-induced phosphorylation of phospholamban, showing that L798106 inhibits the cAMP-decreasing effect of PGE_2.

Data information: Values are mean ± s.e.m. In (A) and (C), n = 4, technical replicates; curve was fitted and half maximal inhibitory concentration (IC_{50} = 27 nM) was determined using GraphPad Prism. In (B), n = 9, independent experiments. *P < 0.05 vs. control, Student's two-tailed unpaired t-test (PE, P = 0.0008; PGE_2, P = 0.0005). The exact n and P-values can also be found in the Source Data Excel file for Fig EV1.
Source data are available online for this figure.

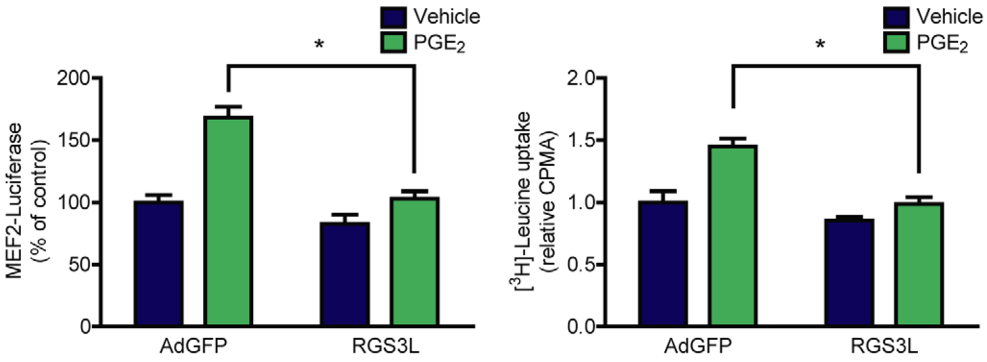

Figure EV2. The PGE_2-induced MEF2 activity and protein synthesis is abolished by overexpression of the βγ-scavenger RGS3L.

Left panel: NRVMs were infected on the first day with 3xMEF2-Luc, on the next day with adenovirus encoding EGFP or RGS3L, then were serum starved. 24-h vehicle or 1 μM PGE_2 stimuli were used. Overexpression RGS3L prevented the PGE_2-induced response. Right panel: After infection with adenovirus encoding EGFP or RGS3L, NRVMs were starved, then treated with vehicle or 1 μM PGE_2 in media containing 1 μCi/ml [^{3}H]-leucine for 24 h. Thereafter, [^{3}H]-leucine uptake was assessed. RGS3L counteracted the effect of PGE_2. Values are mean + s.e.m., n = 3–4, technical replicates, two-way ANOVA, *means significant interaction between RGS3L overexpression and the effect of PGE_2, $P < 0.05$ (MEF2 activity: $P = 0.007$, [^{3}H]-leucine uptake: $P = 0.0393$). The exact n and P-values can also be found in the Source Data Excel file for Fig EV2.

Source data are available online for this figure.

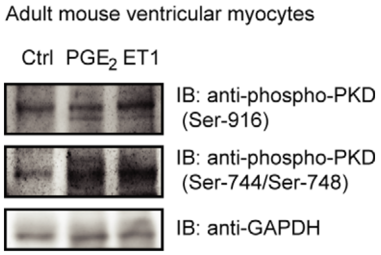

Figure EV3. PGE_2 triggers phosphorylation of PKD in adult mouse ventricular myocytes.

Adult mouse ventricular myocytes were serum starved for 4 h and treated with 1 μM PGE2 or 100 nM ET1 for 4 h. Phosphorylation of PKD was assessed by immunoblot. Representative images are shown.

Source data are available online for this figure.

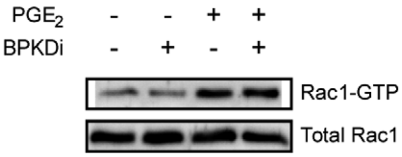

Figure EV4. PGE_2-induced Rac1 activation does not involve PKD.

Serum-starved NRVMs were treated with 3 μM BPKDi prior to stimulation. After treatment with 10 μM PGE_2 for 2 min, Rac1 activation was determined by effector pull-down assay. Representative image from three independent experiments.

Source data are available online for this figure.

© 2018 The Authors
