# Supplementary figures and images for: Inflammation leads through PGE/EP 3 signaling to HDAC5/MEF2‐dependent transcription in cardiac myocytes
Source: EMBO Mol Med. 2018 Jun 15;10(7):e8536. doi: 10.15252/emmm.201708536 (PMC6034133; doi:10.15252/emmm.201708536)

Original blots from Expanded View Figure 1D

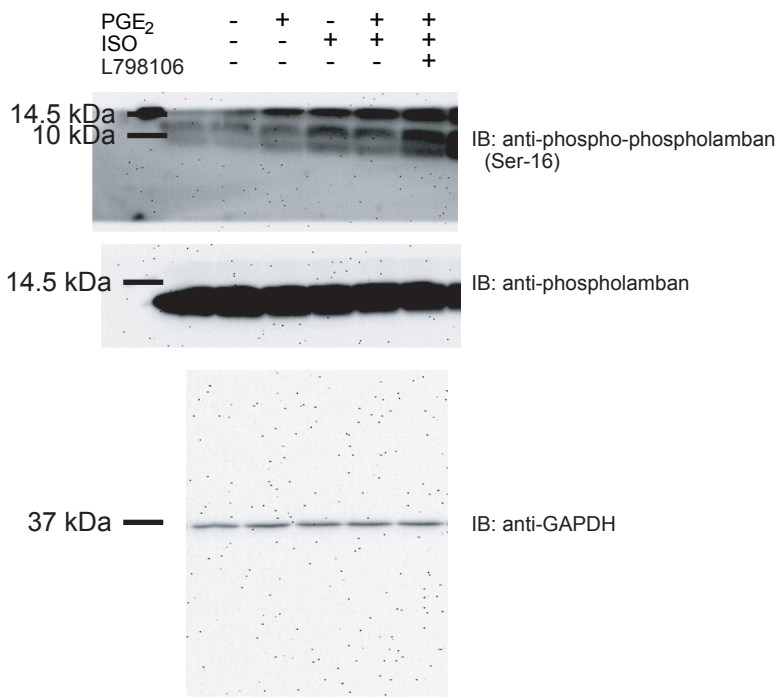

Supplement: Supplementary file 3 — Source Data for Expanded View [file EMMM-10-e8536-s010.zip › emmm201708536-sup-0010-SDataFigEV/SDataFigEV1.pdf]

Original blots from Expanded View Figure 3

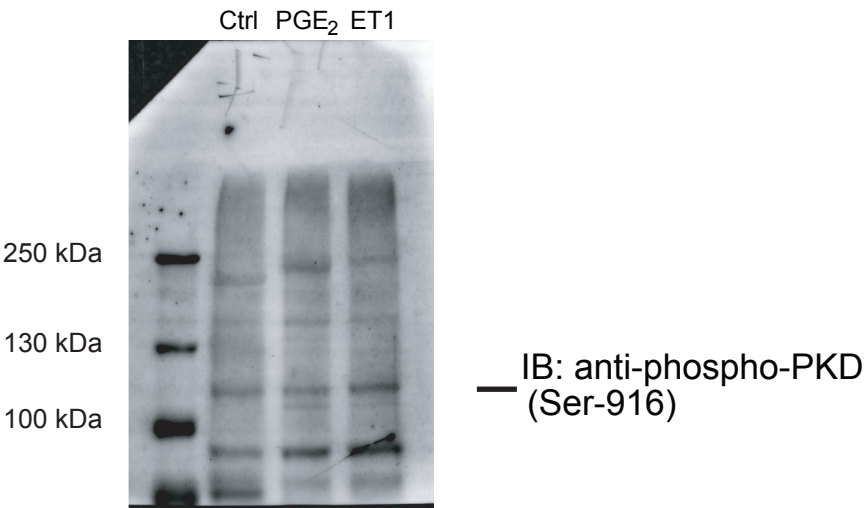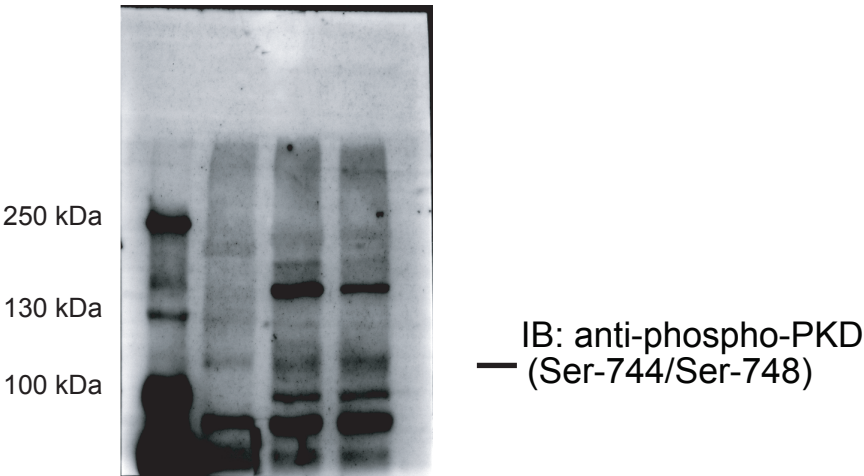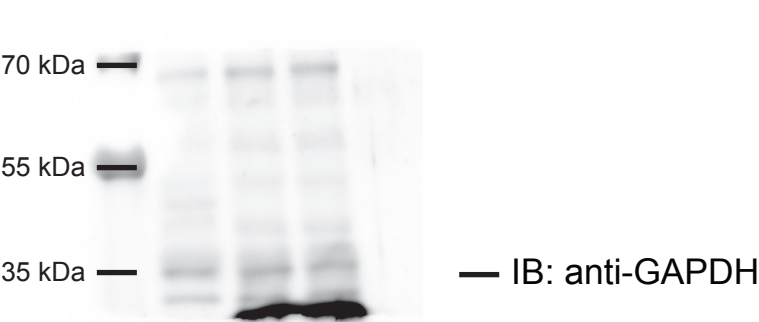

Supplement: Supplementary file 3 — Source Data for Expanded View [file EMMM-10-e8536-s010.zip › emmm201708536-sup-0010-SDataFigEV/SDataFigEV3.pdf]

Original blots from Expanded View Figure 4

|                  | Rac1-GTP |   |   |   | Total Rac1 |   |   |   |
|------------------|----------|---|---|---|------------|---|---|---|
| PGE <sub>2</sub> | -        | - | + | + | -          | - | + | + |
| BPKDi            | -        | + | - | + | -          | + | - | + |

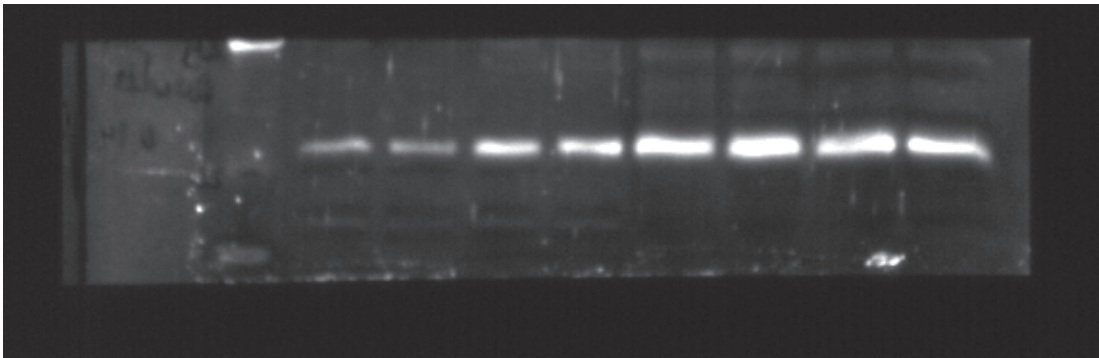

Supplement: Supplementary file 3 — Source Data for Expanded View [file EMMM-10-e8536-s010.zip › emmm201708536-sup-0010-SDataFigEV/SDataFigEV4.pdf]

Original blots from Figure 7D

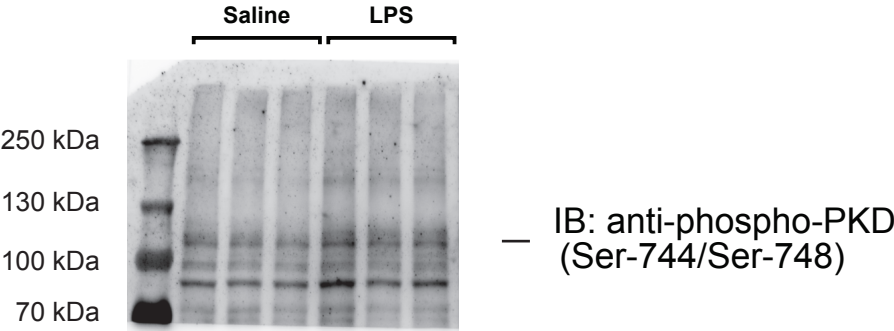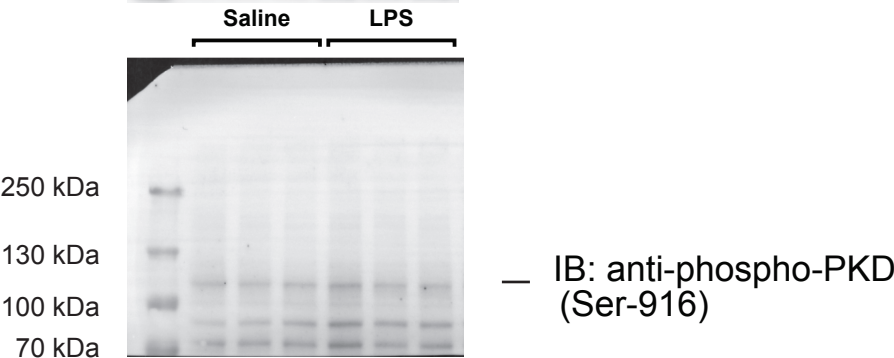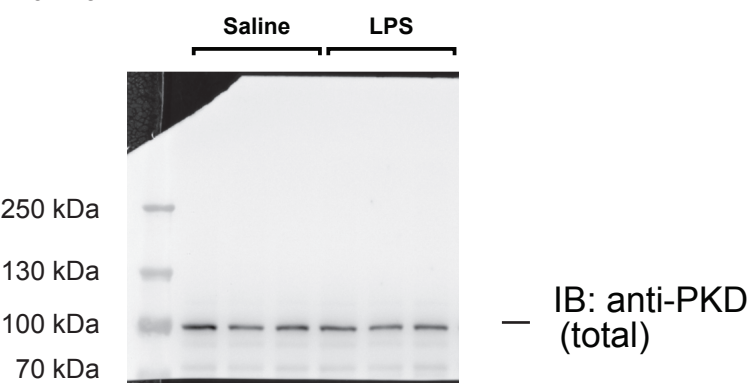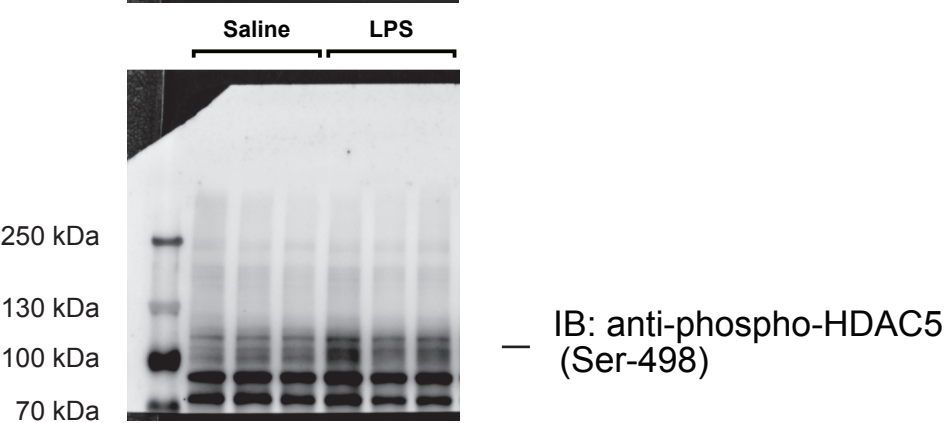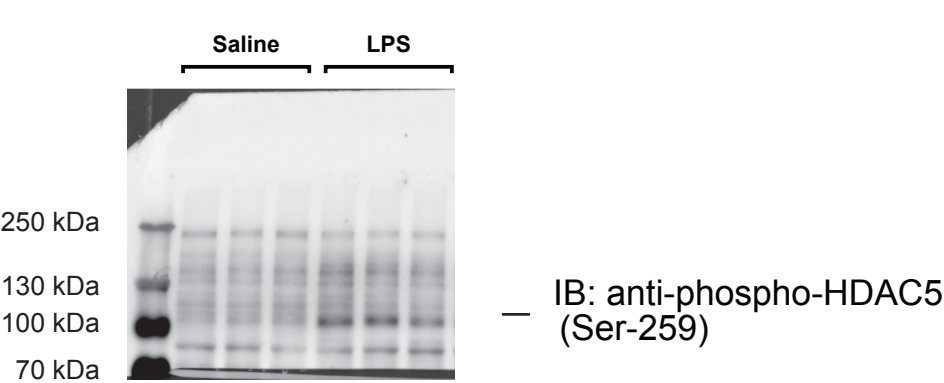

Original blots from Figure 7D

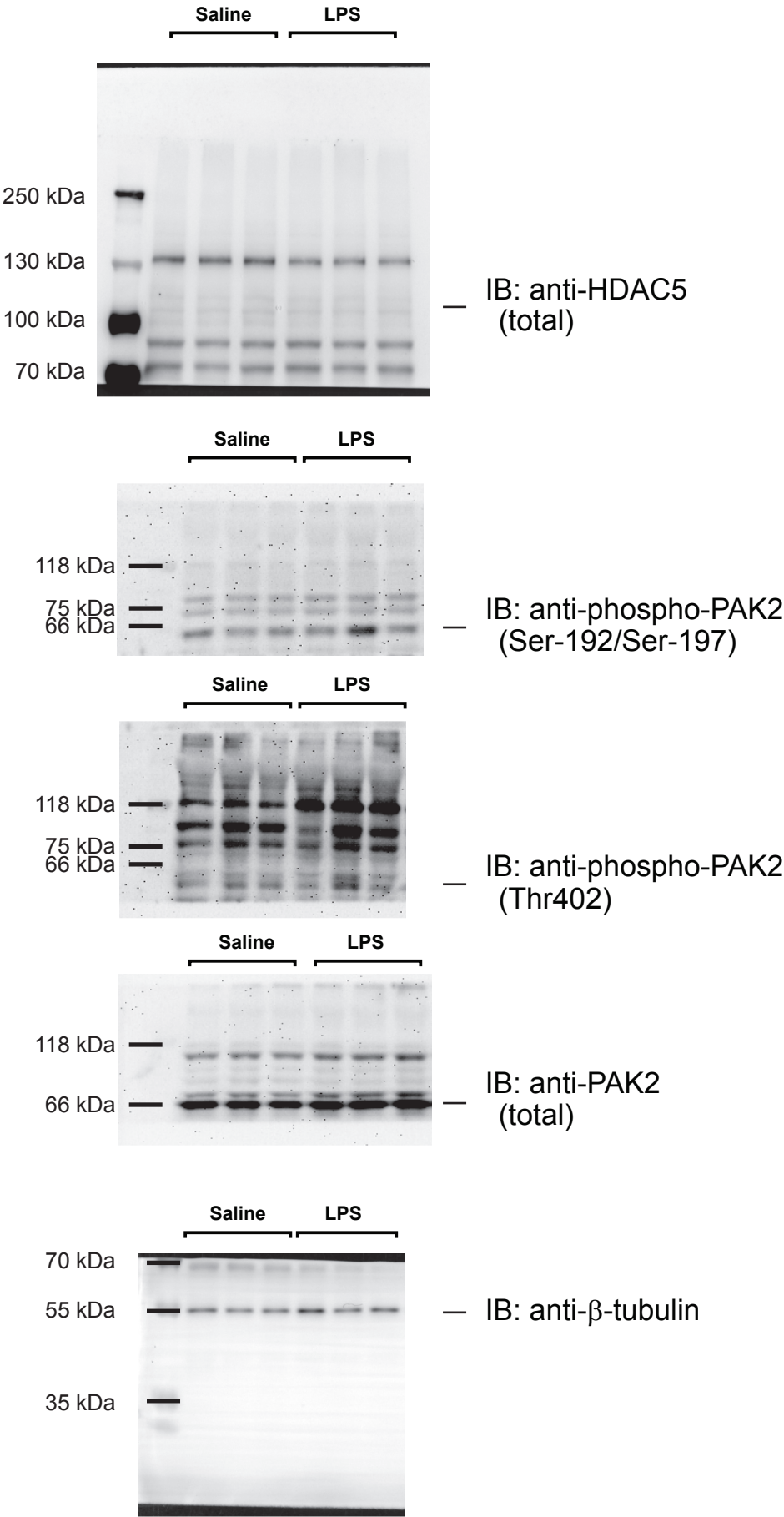

Supplement: Supplementary file 11 — Source Data for Figure 7 [file EMMM-10-e8536-s009.zip › EMM-2017-08536_SourceDataForFigure7.pdf]
