# Supplementary material for: Inflammation leads through PGE/EP 3 signaling to HDAC5/MEF2‐dependent transcription in cardiac myocytes
Source: EMBO Mol Med. 2018 Jun 15;10(7):e8536. doi: 10.15252/emmm.201708536 (PMC6034133; doi:10.15252/emmm.201708536)

Original blots from Figure 3A

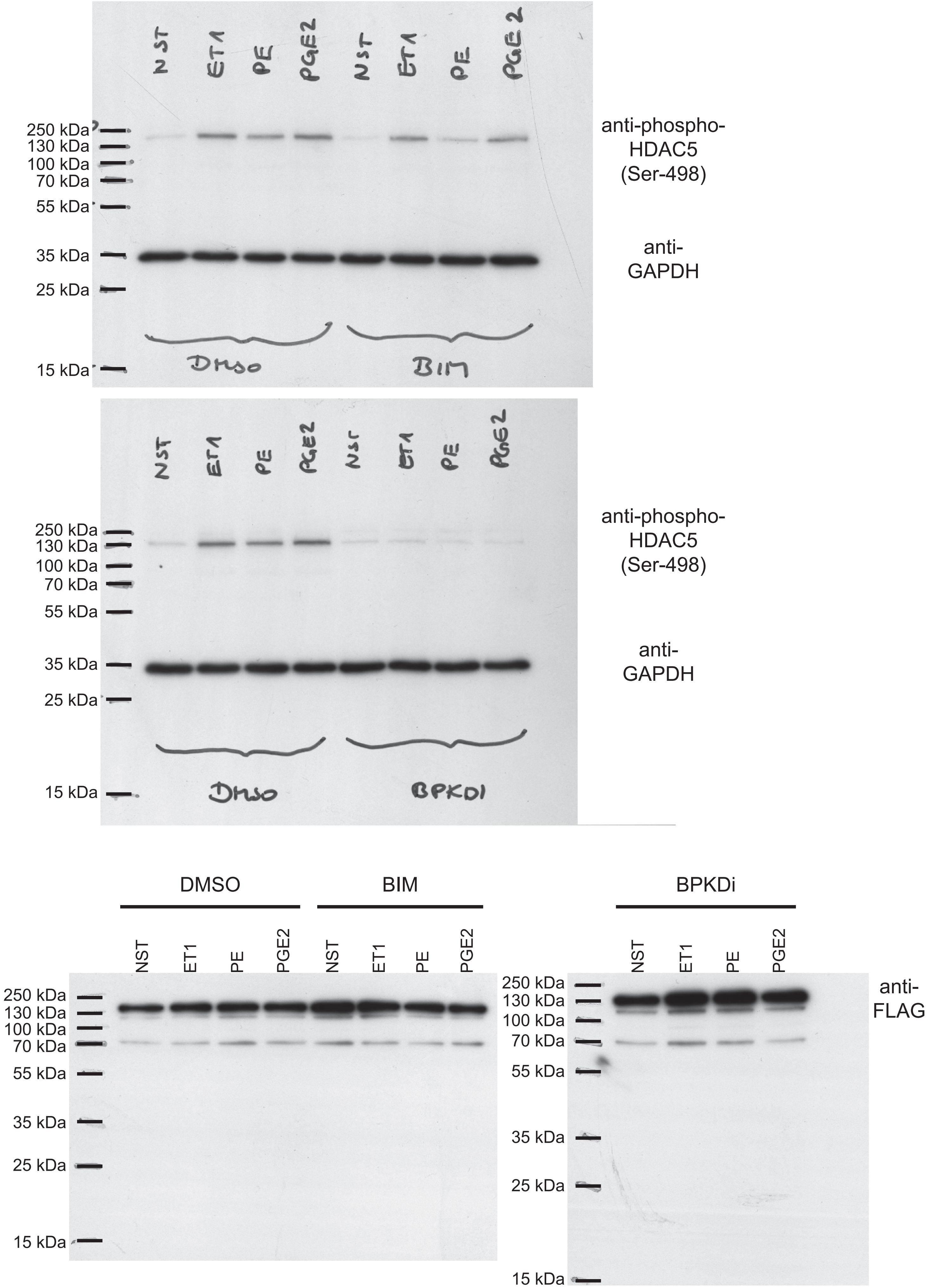

Original blots from Figure 3B

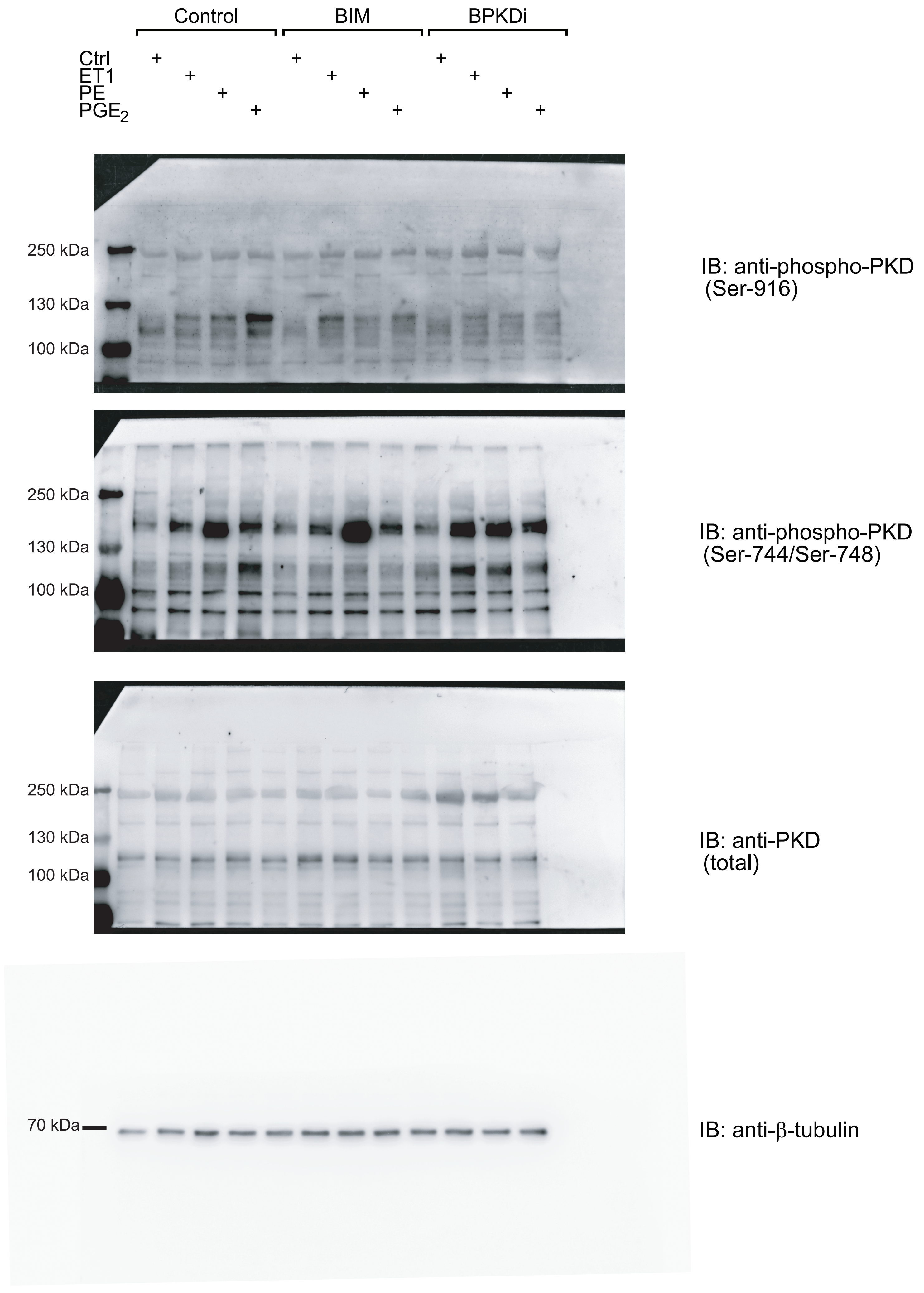

Supplement: Supplementary file 7 — Source Data for Figure 3 [file EMMM-10-e8536-s005.zip › EMM-2017-08536_SourceDataForFigure3.pdf]
