# Supplementary material for: Inflammation leads through PGE/EP 3 signaling to HDAC5/MEF2‐dependent transcription in cardiac myocytes
Source: EMBO Mol Med. 2018 Jun 15;10(7):e8536. doi: 10.15252/emmm.201708536 (PMC6034133; doi:10.15252/emmm.201708536)

Original blots from Figure 4A

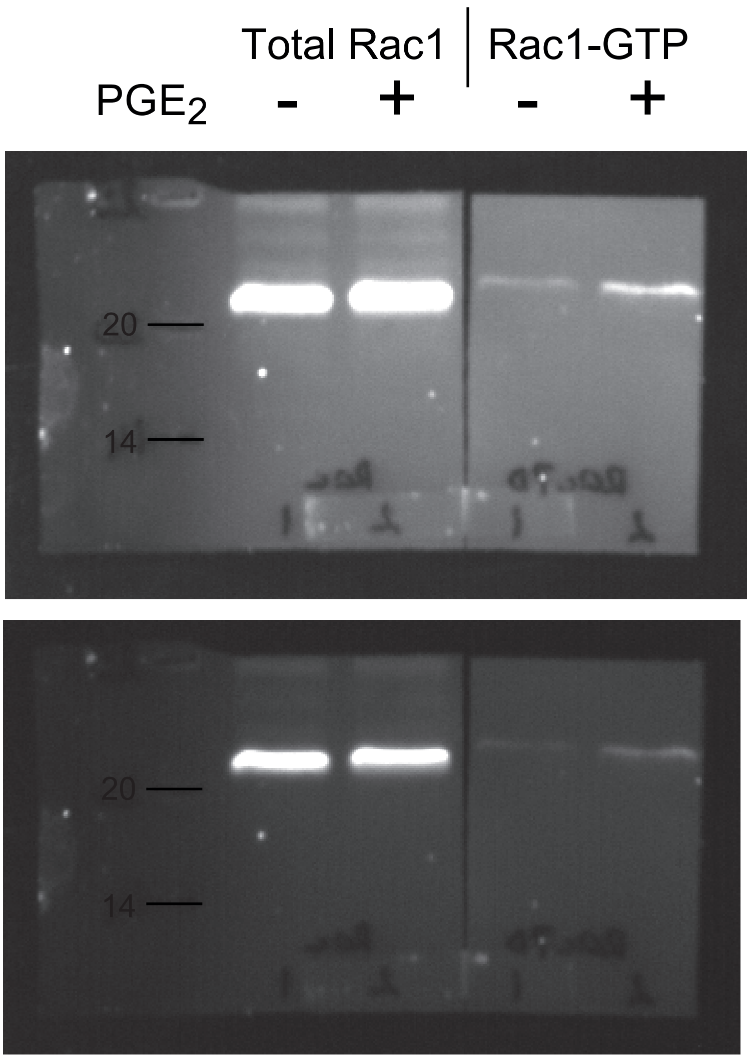

Lower exposure

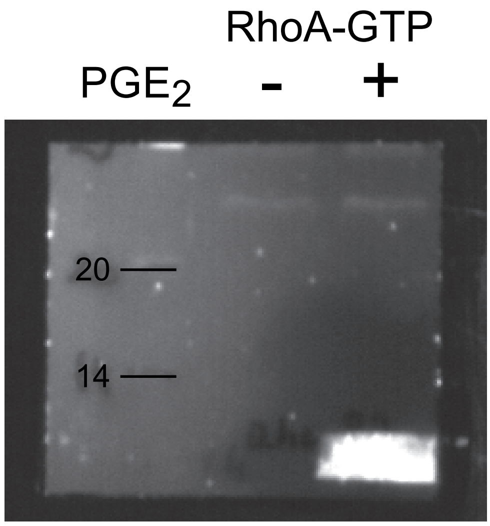

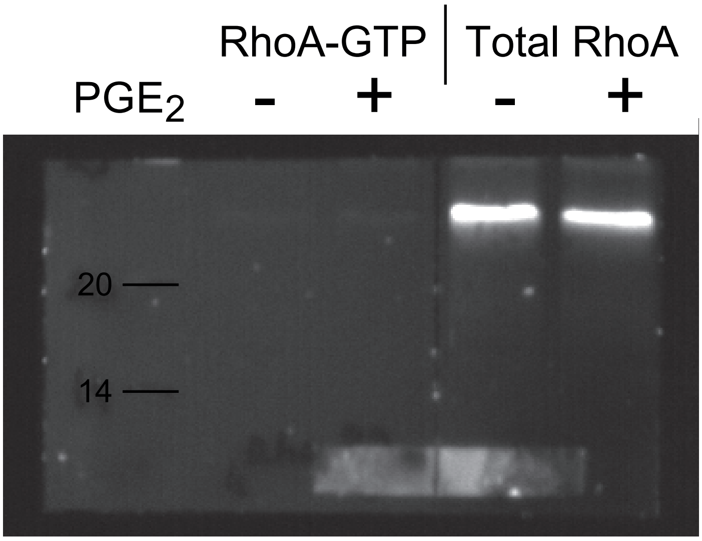

Lower exposure

Original blots from Figure 4B

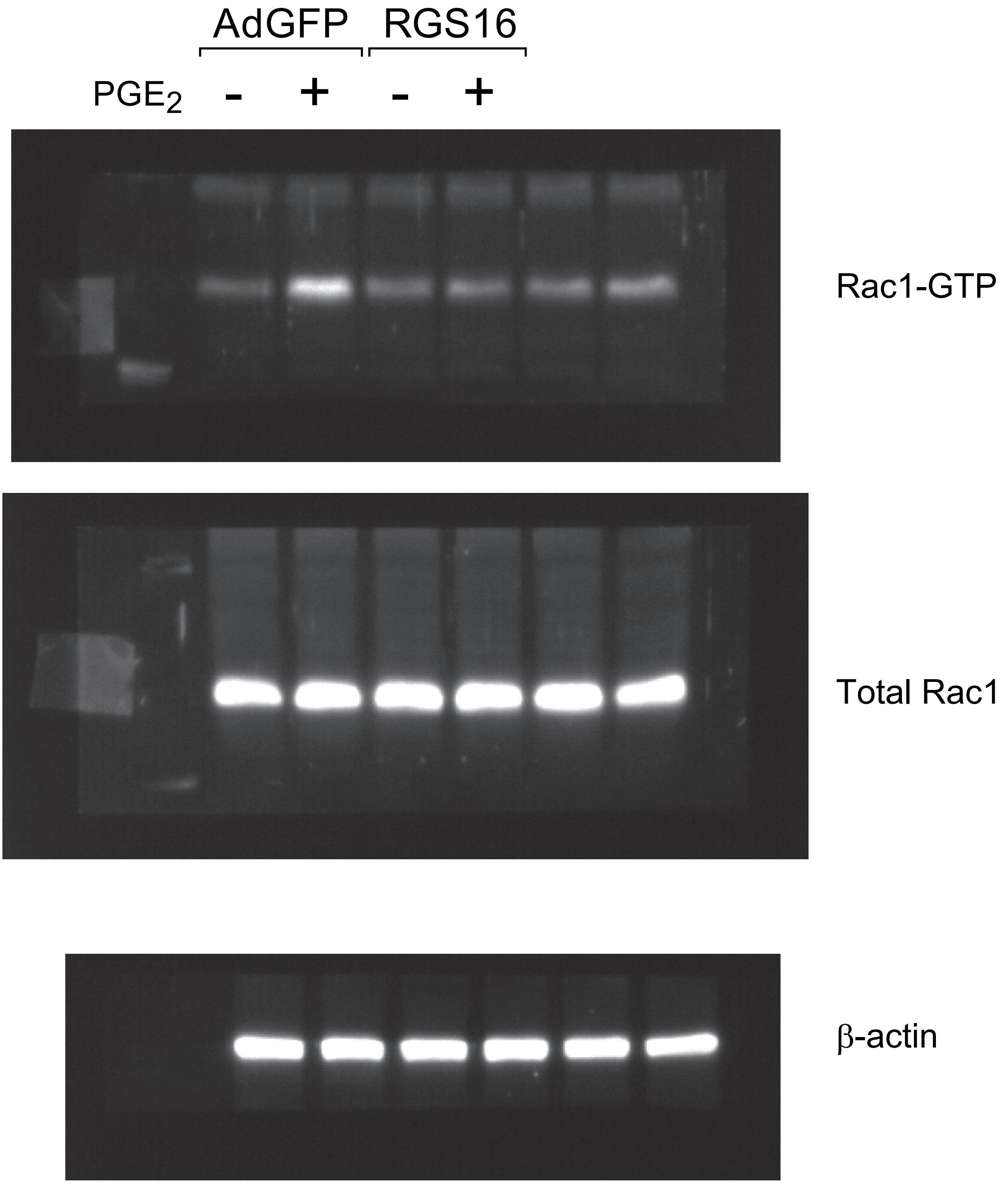

Original blots from Figure 4C

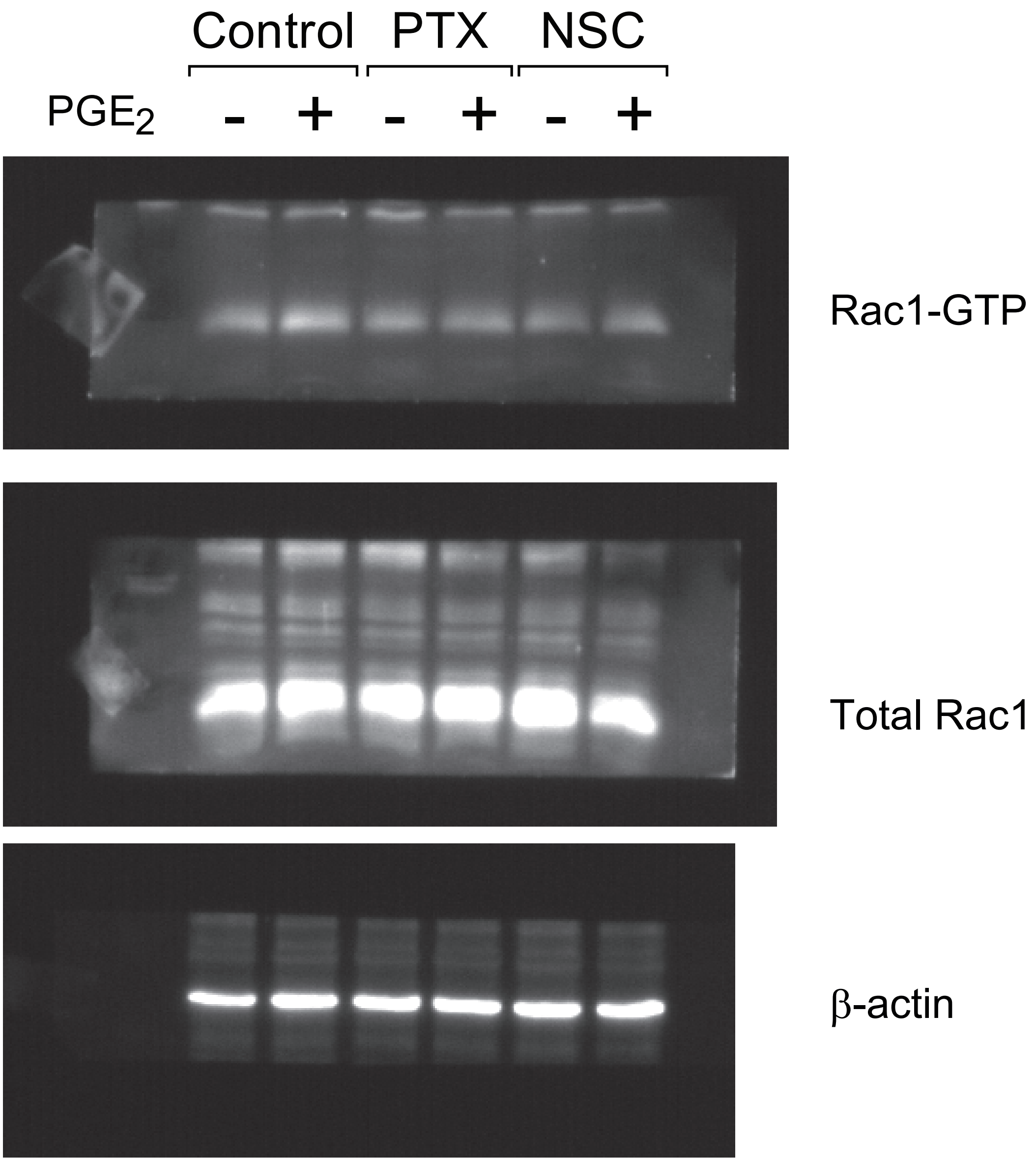

Supplement: Supplementary file 8 — Source Data for Figure 4 [file EMMM-10-e8536-s006.zip › EMM-2017-08536_SourceDataForFigure4.pdf]
