# Supplementary material for: Inflammation leads through PGE/EP 3 signaling to HDAC5/MEF2‐dependent transcription in cardiac myocytes
Source: EMBO Mol Med. 2018 Jun 15;10(7):e8536. doi: 10.15252/emmm.201708536 (PMC6034133; doi:10.15252/emmm.201708536)

Original blots from Figure 5A

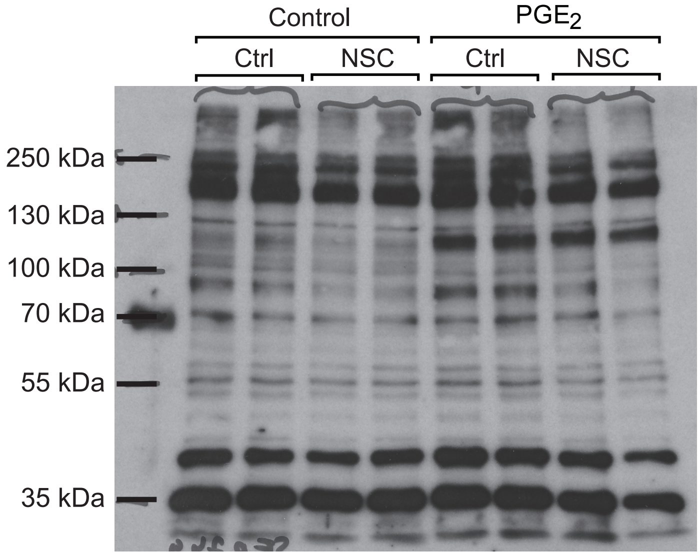

anti-phospho-PKD
(Ser-744/Ser-748)

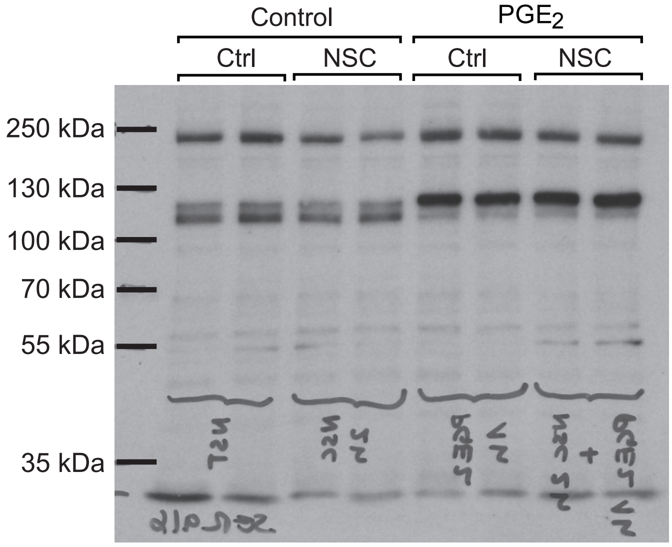

anti-phospho-PKD
(Ser-916)

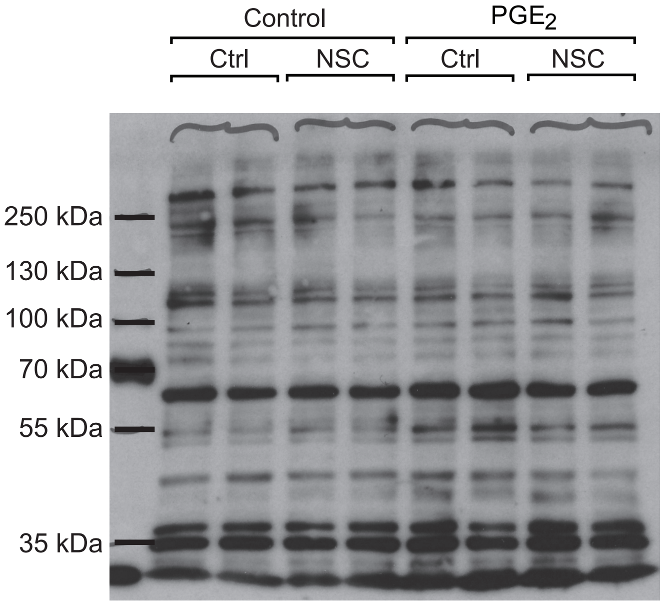

anti-PKD
(total)

Original blots from Figure 5B

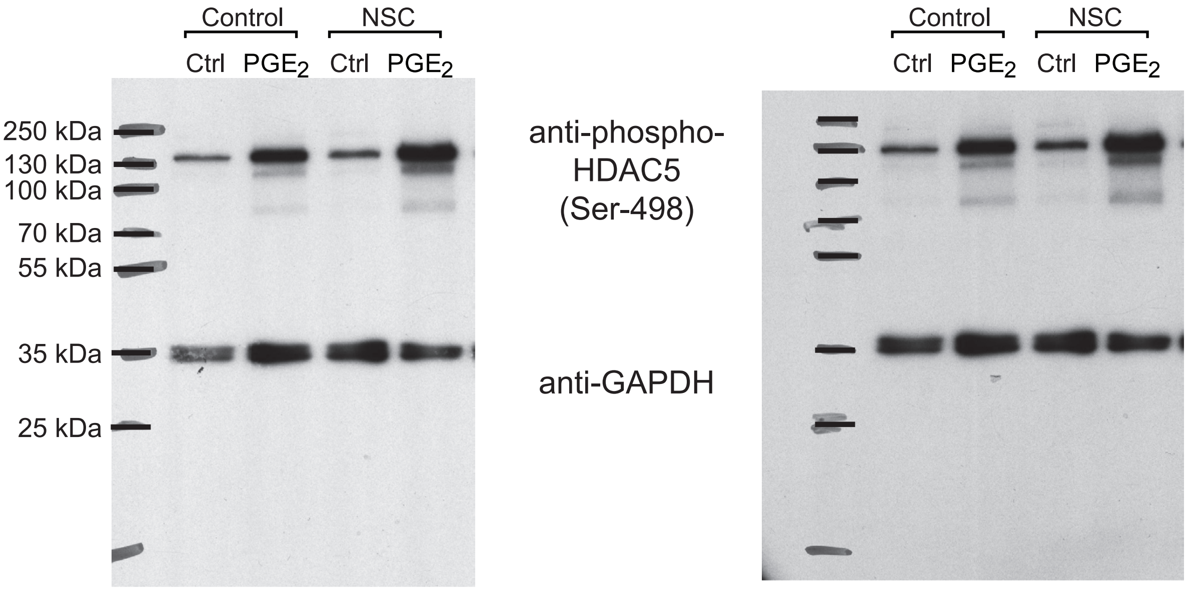

Low exposure

High exposure

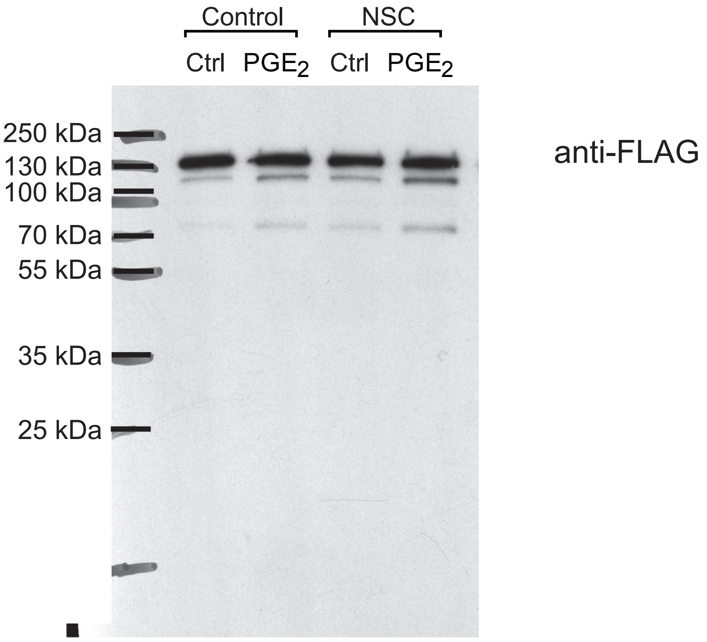

Original blots from Figure 5C

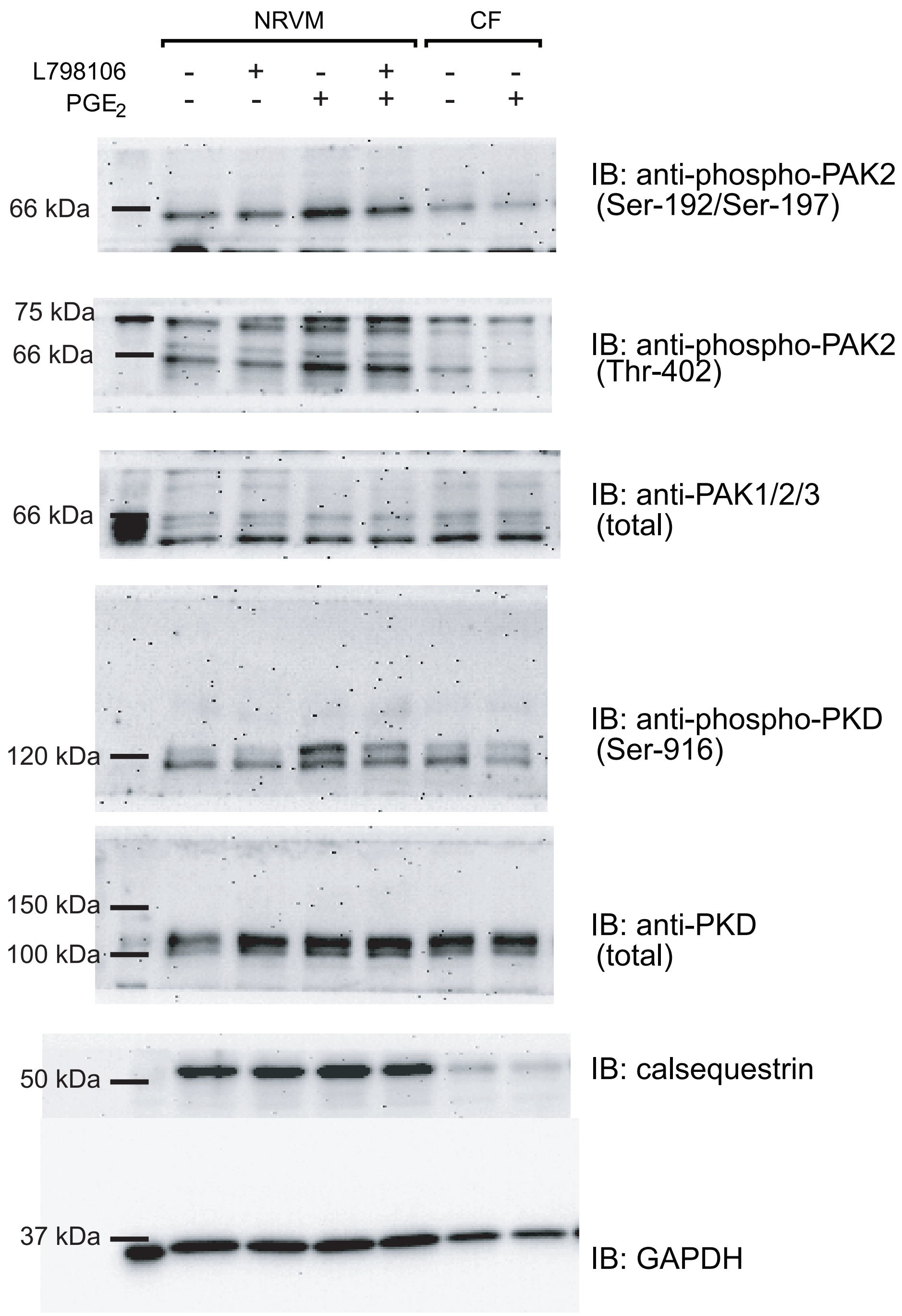

Supplement: Supplementary file 9 — Source Data for Figure 5 [file EMMM-10-e8536-s007.zip › EMM-2017-08536_SourceDataForFigure5.pdf]
